# Pylephlebitis: a rare complication of an intra-abdominal infection

**DOI:** 10.3402/jchimp.v3i2.20732

**Published:** 2013-07-05

**Authors:** Katherine Wong, David S. Weisman, Kelly-Ann Patrice

**Affiliations:** 1 Ross University School of Medicine, North Brunswick, NJ, USA; 2 Good Samaritan Hospital, Department of Medicine, Baltimore, MD, USA

**Keywords:** pylephlebitis, portal vein thrombosis, abdominal infection, sepsis, diverticulitis, appendicitis, bacteroides

## Abstract

Pylephlebitis is defined as an inflamed thrombosis of the portal vein. It is a rare complication of an intra-abdominal infection, and the diagnosis is often missed due to its nonspecific clinical presentation. Symptoms may include abdominal pain, fever, chills, fatigue, nausea, and vomiting. It is important to consider this differential when a patient presents with signs of abdominal sepsis since it has a high mortality rate and is often diagnosed postmortem. Pylephlebitis can be diagnosed via abdominal ultrasound or CT demonstrating a thrombus in the portal vein, and it must be treated early and aggressively with broad-spectrum antibiotics. We are presenting a case of pylephlebitis as well as discussing the diagnosis and treatment of this potentially lethal condition.

Pylephlebitis is defined as a suppurative and inflamed thrombosis of the portal vein (1), which is a rare but potentially lethal complication of an intra-abdominal infection with a mortality rate of 25%. This condition can be difficult to diagnose due to its nonspecific clinical presentation. However, it can be treated with early and aggressive intervention. It is important to keep this diagnosis on the differential of abdominal pain as it is associated with many common intra-abdominal pathological conditions and carries with it a high morbidity and mortality rate.

## Case report

A 56-year-old African–American male presented to the emergency room with a chief complaint of fever, chills, and weakness. He was in his usual state of health until one week prior to presentation, when he began feeling chills and ill-defined periumbilical pain. The patient also reported experiencing dizziness, headache, and decreased appetite. He denied any recent travel or sick contacts.

The patient's past medical history included diabetes mellitus type II, hypertension, hyperlipidemia, cellulitis, and deep venous thrombosis (DVT) treated with enoxaparin for 6 months. The DVT occurred approximately 5 years ago, and he could not recall whether it was provoked. He denied having a hypercoagulable workup done in the past, and he denied any family history of DVT or any hypercoagulable states. Home medications included hydrochlorothiazide, metformin, and simvastatin. The patient was working as a custodian, and he denied any history of alcohol, tobacco, or illicit drug use..

The patient's vital signs on presentation were: T 36°C, BP 144/108, HR 78, RR 36, and SpO2 93% on room air. His examination was unremarkable with the exception of mild tenderness on deep palpation of the lower abdomen, but no guarding or rebound tenderness. The spleen and liver were non-palpable.

Lab studies revealed leukocytosis (18,900 cells/mL, reference range 4,500–10,000 cells/mL) and anemia (Hb 12.3, Hct 37.2, MCV 78.3). INR was 1.5 and amylase was normal (40 units/L, reference range 30–110 units/L). Liver panel showed an increased AST (94 units/L, reference range 3–34 units/L) and ALT (104 units/L, reference range 15–41 units/L) with an elevated total bilirubin of 1.8 mg/dL (reference range 0.2–1.3 mg/dL). Urinalysis was unremarkable. Lactic acid was elevated (2.7 mmol/L, reference range 0.5–2.2 mmol/L).

**Fig. 1:**
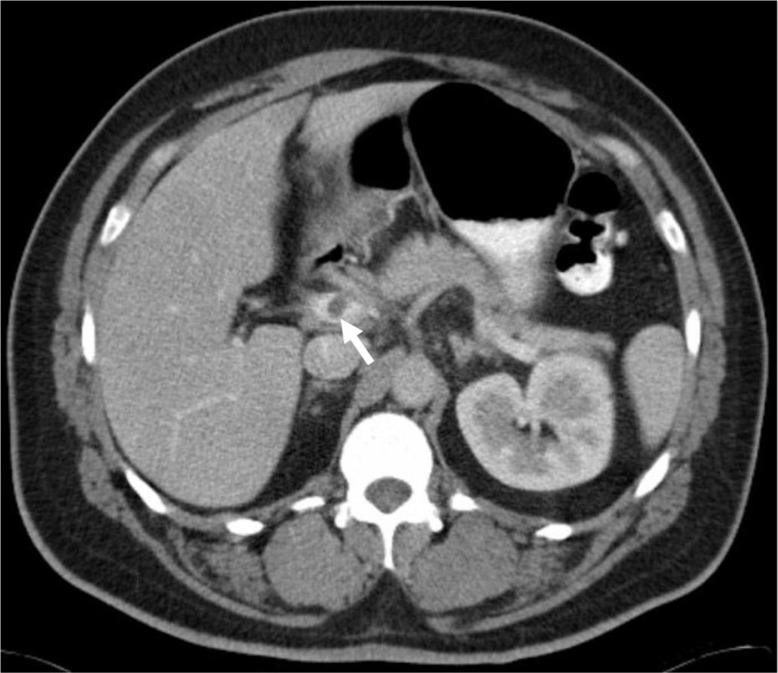
Contrast enhanced CT of the abdomen showing a nonocclusive thrombus within the main portal vein (white arrow).

**Fig. 2:**
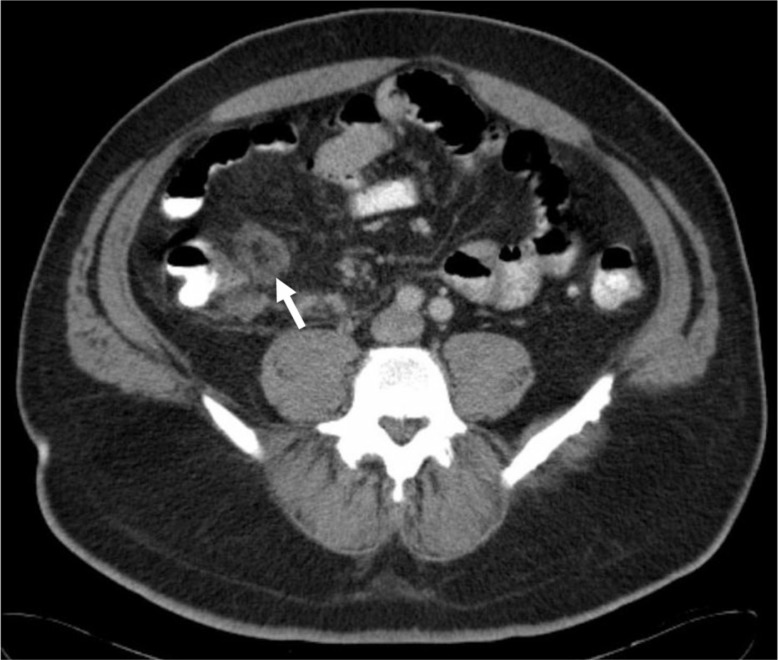
Contrast enhanced CT of the abdomen showing a focal 3.2-cm mass (white arrow) adjacent to the ileocecal valve along the mesenteric border with central fat attenuation and surrounding soft tissue attenuation suggesting epiploic appendicitis or a fatty infarct.

An abdominal and pelvic CT scan was completed to rule out the possibility of appendicitis, diverticulitis, or an intra-abdominal abscess. The CT revealed a non-occlusive thrombus within the main portal vein that extended throughout the superior mesenteric vein (SMV) (Fig. 1). An ill-defined, non-enhancing 3.2 cm mass concerning for appendicitis or fatty infarct was also discovered in the ileocecal region of the right lower quadrant (Fig. 2). There were no hepatic abnormalities and there was no evidence of diverticulitis or abscess.

A heparin drip was administered for the SMV thrombus. The patient was also empirically started on vancomycin and piperacillin/tazobactam for sepsis.

**Fig. 3:**
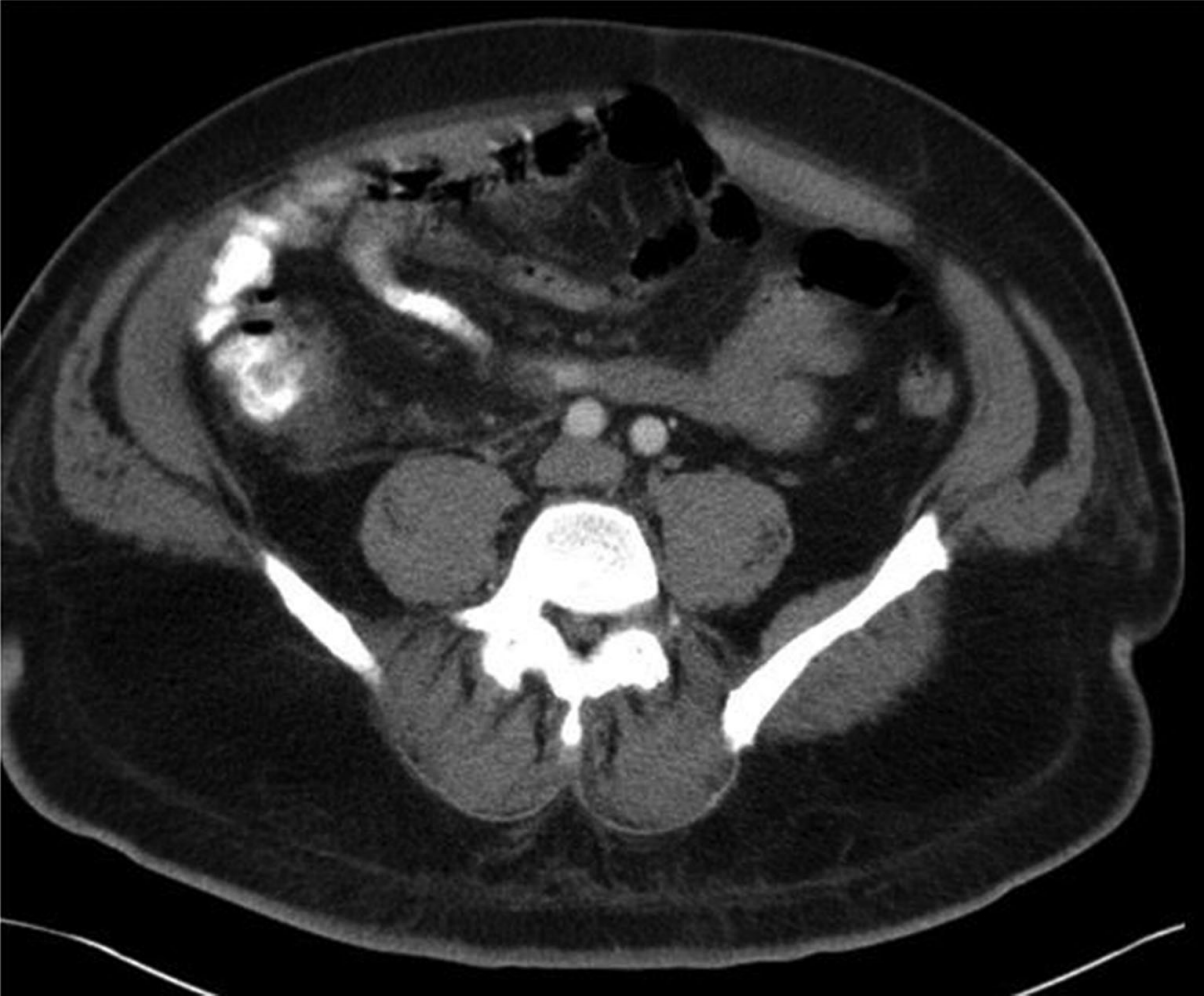
Contrast-enhanced CT of the abdomen on hospital day 7 showing resolution of the ileocecal mass.

On day two of hospitalization, the patient reported improving symptoms and his abnormal labs also improved. He was evaluated by a surgeon, and it was decided that emergent surgery was not needed since his CT scan and physical exam findings were not typical for appendicitis. A gastroenterologist also performed a colonoscopy which was unremarkable. Blood cultures grew *Escherichia coli* and *Bacteroides fragilis* on day four, and vancomycin was replaced with metronidazole for *Bacteroides coverage*. A repeat blood culture was obtained on hospital day seven which did not exhibit any growth. A follow-up CT scan of the abdomen demonstrated resolution of the ileocecal mass (Fig. 3).

The patient was discharged 10 days after admission on warfarin and ertapenem (the latter for 4-week duration). Four months after hospitalization, the patient was feeling well. He finished his antibiotic course was continuing on anticoagulation.

## Discussion

The most commonly reported etiology of pylephlebitis is diverticulitis, followed by appendicitis, cholecystitis, pancreatitis, and other intra-abdominal infections (2, 3). A recent abdominal surgery can also predispose to pylephlebitis (3). However, in a study done by Waxman *et al*., they found that a primary source of infection could not be identified in 70% of patients (4). There is also little data to support any inherited coagulopathies as an underlying etiology (3). There is no greater predisposition with age, although males have a slightly higher predominance (68-74%) (3, 5).

Pylephlebitis occurs as a result of an abdominal infection draining into the portal venous system. The infection is usually polymicrobial, and *Bacteroides fragilis* is the most commonly isolated organism (6). It facilitates coagulation via its surface and capsular components. The surface component accelerates fibrin cross-linking and the capsular polysaccharides initiate the clotting cascade by activating macrophages (7). Other organisms that have been isolated in patients with pylephlebitis include *Escherichia coli*, *Proteus mirabilis*, *Clostridium* species, *Klebsiella, Pneumococcus, Aerobacter*, and *Streptococcus* species (3, 6, 8).

The symptoms of pylephlebitis are non-specific and the condition is potentially lethal. There should be a high clinical suspicion in patients who present with abdominal pain, fever, and other signs of sepsis, as well as leukocytosis and elevated liver enzymes (8, 9). Other common clinical features include fatigue, malaise, chills, nausea, vomiting, diarrhea, and anorexia/weight loss (3, 8). Patients may also have hepatomegaly and jaundice. The abdominal exam is often unrevealing of the infection source since the pain severity can range from mild to severe and location can be focal or diffuse (8, 10).

Pylephlebitis can be diagnosed via abdominal ultrasonography showing a thrombus in the portal vein (5). An abdominal CT scan is less operator-dependent and is more widely used because of its ability to detect other sources of infection in the abdomen. The sensitivity and specificity of both ultrasound and CT scan imaging in diagnosing pylephlebitis are not known; however, the quality of an ultrasound will vary based on the operator, and a CT scan will depend on the image quality as well as the skill of the reader to detect the more subtle findings (9). Positive blood cultures are found in 50–88% of patients (5, 11). Lab tests are usually nonspecific, but the most common abnormal labs include leukocytosis, elevated AST/ALT, and anemia (3).

If pylephlebitis is suspected, broad-spectrum antibiotics that cover Gram-negative bacilli, anaerobes, and aerobes should be administered immediately and subsequently modified pending culture results (9). An empiric antibiotic regimen has not been established, but successful therapies have included metronidazole, gentamicin, piperacillin, ceftizoxime, imipenem, and ampicillin (5, 8, 12). A standard duration of antibiotic therapy has not been established either, although it has been reported that antibiotics should be administered for a minimum of 4 weeks to prevent development of a hepatic abscesses, which is a commonly reported complication (5).

Due to the limited data available, there has been much debate over the role of anticoagulation and whether it is essential in the treatment of pylephlebitis. It has been proposed that the purpose of anticoagulation in pylephlebitis is to prevent bowel ischemia and infarction secondary to extension of the thrombus (3, 13). In the retrospective case series studies done by Plemmons et al. and Kanellopoulou et al., it was found that patients who received both antibiotics and heparin had a better outcome than those who only received antibiotics (3, 5). However, in an expert opinion derived from case report(s) done by Kasper *et al*. anticoagulation did not result in better outcomes in pylephlebitis over antibiotics alone (6). In a retrospective case series study done by Baril *et al*. it was concluded that only patients who had clotting factor deficiencies (protein S deficiency, protein C deficiency, antithrombin III deficiency, factor XII deficiency, or anti-cardiolipin antibodies) or neoplasms resulting in a hypercoagulable state would need anticoagulation (10). In an expert opinion case report done by Duffy *et al*. anticoagulation was recommended if the thrombosis extended beyond the portal vein and/or was causing ischemia. It was also indicated if the patient was not responding to antibiotics, surgical intervention, or both. However, anticoagulation was not recommended if the patient had a portal vein thrombus but did not have any systemic manifestations, or if he or she had a chronic pylethrombosis (12). There has not been an established consensus on the duration of anticoagulation therapy in pylephlebitis (10).

The role of thrombolytics in the treatment of pylephlebitis is also not well-known. In a case report by Sherigar *et al*. it was concluded that the indication for thrombolytic therapy was similar to that of using anticoagulation: to prevent the progression of the thrombus as well as portal hypertension, which is a complication associated with higher mortality. Therefore, tPA could be a consideration if the patient was unresponsive to antibiotics indicating a persistent infection (14).

The treatment of pylephlebitis can also involve drainage of a focus of infection, such as a hepatic or pericolonic abscess (5). A liver abscess smaller than 3 cm can be treated with antibiotics alone, but an abscess larger than 3 cm will require drainage percutaneously (9). Surgery on the thrombosed and infected vessels is no longer widely practiced (15) due to a higher risk of recurrence of the thrombosis (3), although it can be done in patients who are not responsive to antibiotic and anticoagulation therapy (16).

Mortality in patients with pylephlebitis is more likely due to severe sepsis secondary to an overwhelming intra-abdominal infection rather than the thrombosis leading to bowel infarction (3, 5). The reported mortality rates have been high, ranging from 50% to 80% with many cases being diagnosed postmortem (9, 17). However, in recent years the mortality rate has decreased to 25% due to earlier detection through imaging and aggressive antibiotic therapy (3).

## Conclusion

Pylephlebitis is an uncommon complication of an intra-abdominal infection, with diverticulitis being the most common etiology. Due to the high mortality rate, there should be a high clinical suspicion for patients who present with signs of abdominal sepsis and have non-specific lab findings after other etiologies have been excluded. The mainstay of treatment is aggressive antibiotic therapy, and the routine use of anticoagulation continues to be debated. Mortality has decreased to 25% in recent years through earlier diagnosis with imaging and appropriate therapy.
